# Association of the del443ins54 at the *ARMS2* locus in Indian and Australian cohorts with age-related macular degeneration

**Published:** 2013-04-05

**Authors:** Inderjeet Kaur, Stuart Cantsilieris, Saritha Katta, Andrea J. Richardson, Maria Schache, Rajeev R. Pappuru, Raja Narayanan, Annie Mathai, Ajit B. Majji, Nicole Tindill, Robyn H. Guymer, Subhabrata Chakrabarti, Paul N. Baird

**Affiliations:** 1 Hyderabad Eye Research Foundation, L.V. Prasad Eye Institute, Hyderabad, India; 2 Centre for Eye Research Australia, University of Melbourne, Royal Victorian Eye and Ear Hospital, Melbourne, Victoria, Australia; 3 Hyderabad Eye Institute, L.V. Prasad Eye Institute, Hyderabad, India

## Abstract

**Purpose:**

The *ARMS2/HTRA1* genes at the 10q26 locus have been associated with risk of age-related macular degeneration (AMD), with the most significantly associated variants being A69S (rs10490924), del443ins54 (EU427539) and rs11200638. We wished to explore the association of the del443ins54 in two ethnically different populations from India and Australia.

**Methods:**

The del443ins54 was screened in a large cohort of ~1500 subjects from these two populations by a combination of PCR-based agarose gel electrophoresis and validated by resequencing. Statistical analysis comprised the calculations of allele, genotype and haplotype frequencies along with their p values and corresponding odds ratios (OR), and 95% confidence intervals (95% CI) and measures of linkage disequilibrium (LD).

**Results:**

The del443ins54 was significantly associated with AMD in both the Indian (p=1.74×10^−13^; OR=2.80, 95%CI, 2.12–3.70) and Australian cohorts (p=2.78×10^−30^; OR=3.15, 95%CI, 2.58–3.86). These associations were similar to those previously identified for the A69S and the rs11200638 variant in these populations that also exhibited high degrees of LD (D’ of 0.87-0.99). A major risk haplotype of “T-indel-A” (p=5.7×10^−16^; OR=3.16, 95%CI, 2.34–4.19 and p=6.33×10^−30^; OR=3.15, 95%CI, 2.57–3.85) and a protective haplotype of “G-wild type-G” (p=2.35×10^−11^; OR=0.39, 95%CI, 0.29–0.52 and p=1.02×10^−30^; OR=0.31, 95%CI, 0.25–0.38) were identified in the Indian and Australian cohorts, respectively.

**Conclusions:**

These data provide an independent replication of the association of del443ins54 variant in two different ethnicities, despite differences in allele and haplotype frequencies between them. High levels of LD in both populations limit further genetic dissection of this region in AMD.

## Introduction

Age-related macular degeneration (AMD) is a complex multifactorial disease and a leading cause of irreversible blindness in the world [1]. A major AMD susceptibility locus on 10q26 has been found to harbor risk associated variants in *ARMS2* (rs10490924) and *HTRA1* (rs11200638) in multiple populations worldwide [1-4]. An insertion-deletion (indel) polymorphism (EU427539) that affects the stability of *ARMS2* mRNA by the removal of a polyadenylation signal (443 bases) and insertion of a 54bp AU rich element in the 3′-UTR (del443ins54), has also been identified in the 3′ end of the *ARMS2* gene as increasing risk of AMD by several fold in both Caucasian [5] as well as Asian populations. Previous associations for this indel vary (from p=3.5×10^−5^ to p=8.4×10^−34^) in individuals of European origin [6-9], whereas, this has only been reported in two non-European cohorts consisting of Han Chinese [8] and Japanese populations [10].

We have previously reported a significant association of the A69S (rs10490924) and rs11200638 variants with AMD in both South Indian and Australian cohorts [11,12]. We now wished to determine the risk conferred by del443ins54 and its combined effect with both the A69S and rs11200638 variants with AMD susceptibility in two ethnically different cohorts from Southern India and Australia.

## Methods

The study protocols adhered to the tenets of the Declaration of Helsinki and were approved by the Institutional Review Boards of the Royal Victorian Eye and Ear Hospital, Melbourne, Australia and L.V. Prasad Eye Institute, Hyderabad, India. The del443ins54 was screened in end stage AMD cases (mainly choroidal neovascular) and normal controls from cohorts in India (n=433) and Australia (n=1054). The detailed methods of clinical diagnosis along with the inclusion and exclusion criteria have been previously reported [11,12]. Amplification was performed using forward (5′-TCT GTG CAG CTG GTG AAA TC-3′) and reverse (5′-TCC AGG GTG GTG TAA TCC AT-3′) primers at an annealing temperature of 61 °C. Amplicons were visualized on a 2% agarose gel and genotypes directly scored from the gels. Subsets of samples were further validated by bi-directional sequencing on an automated DNA sequencer (ABI 3100), using the BigDye chemistry as per manufacturer’s guidelines (both from Applied Biosystems, Foster City, CA). Genotyping results were independently validated by a second investigator who was masked to the phenotype data.

Allele and genotype frequencies were determined by the gene counting method and estimates of Hardy–Weinberg equilibrium were assessed. Odds ratios (OR) and 95% confidence intervals (95%CI) were calculated to assess the risk conferred for each variant using the PLINK software [13]. Haplotypes were generated using various combinations of the A69S, the indel and rs11200638 variants and the estimated haplotype frequencies and linkage disequilibrium (LD) were assessed with the Haploview software (version 4.2) that uses the EM algorithm [14].

## Results

All statistical analyses were based on samples where genotyping was successful across all three A69S, del443ins54 and rs11200638 genetic variants. Each variant was in Hardy–Weinberg equilibrium in both the Indian and Australian cohorts (p>0.05). Allele frequencies for each of the risk variants (T allele of A69S, presence of the indel in del443ins54 and the A allele of rs11200638) exhibited a relatively higher frequency (0.60–0.63) in the Indian cohort compared to the European cohorts (0.36–0.53) but lesser than that reported in Han Chinese (0.73–0.77) or the in the Japanese (0.86–0.88) populations (Table 1). The allele frequencies in the Australian cohort were similar (0.44) to those previously reported for other European cohorts (Table 1).

**Table 1:** Risk allele frequencies of the *ARMS2* (A69S and del443ins54) and *HTRA1* SNPs in different populations.

Population (N=Cases, Controls)	rs10490924 (A69S; “T” risk allele)				del443ins54 (**Indel)**				rs11200638 (“A” risk allele)			
	Case	Control	P value	OR (95%CI)	Cases	Controls	P value	OR(95%CI)	Cases	Controls	P value	OR (95%CI)
German (760, 549)^5^	0.424	0.193	2.8 x10^-29^	2.86(2.38-3.44)	0.424	0.193	4.1 x10^-29^	2.85(2.37-3.43)	0.426	0.199	6.9 x 10^-29^	2.85(2.37-3.42)
Caucasian (819, 329)^6^	0.412	0.248	1.89 x 10^-13^	2.13 (1.74-2.61)	0.409	0.248	3.62 x 10^-13^	2.1 (1.71-2.57)	NA	NA	NA	NA
Caucasian (291, 191)^7^	0.36	0.23	3.31 x 10^-5^	1.86	0.36	0.23	3.46 x 10^-5^	1.85	0.36	0.24	6.41 x 10^-5^	1.8
Utah (705, 650)^8^	0.38	0.2	8.61 x 10^-26^	NA	0.39	0.2	1.9 x 10^-26^	NA	0.41	0.22	3.64 x 10^-26^	NA
Northern European (442, 434)^8^	0.52	0.24	4.87 x 10^-34^	NA	0.53	0.25	8.35x10^-34^	NA	0.53	0.25	2.52 x 10^-34^	NA
Italian (159, 286)^9^	NA	NA	NA	NA	0.51	0.24	2.7x10^-15^	3.25 (2.36-4.41)	NA	NA	NA	NA
Han Chinese (138, 591)^8^	0.74	0.49	1.15 x 10^-13^	NA	0.73	0.49	6.03 x 10^-13^	NA	0.77	0.52	5.10 x 10^-13^	NA
Japanese (56, 77)^10^	0.86	0.62	NA	NA	0.875	0.66	NA	NA	NA	NA	NA	NA
Australian (624,430)*	0.445	0.202	1.97x10^-30^	3.14(2.58-3.86)	0.446	0.199	2.78x10^-30^	3.15 (2.58-3.86)	0.441	0.202	1.43x10^-29^	3.11(2.54-3.80)
South Indian (227, 206)*	0.63	0.36	1.85x10^-15^	3.06 (2.31-4.04)	0.63	0.38	1.74x10^-13^	2.8 (2.12-3.70)	0.6	0.35	9.11x10^-11^	2.76 (2.02-3.77)

*Data from the current study ; NA = Data not available

The frequency of the del443ins54 variant was significantly higher among AMD cases than controls in both the Indian (p=1.74×10^−13^; OR=2.80, 95%CI, 2.12–3.70) and Australian (p=2.78×10^−30^; OR=3.15, 95%CI, 2.58–3.86), cohorts (Table 1). This increased risk was also observed with respect to the del443ins54 genotypes. These findings were similar for the A69S (*ARMS2*) and the rs11200638 (*HTRA1*) variants in both the Indian and Australian cohorts, respectively (Table 2).

**Table 2:** Genotype counts of the *ARMS2* and *HTRA1* SNPs in the Indian and Australian cohorts

Population	SNP (gene)	Genotypes	Genotype counts		P value	Odds ratios (95% CI)
			Cases	Controls		
Australian	rs10490924 (*ARMS2*)	GG	189	271	-	Ref
		GT	282	138	<0.0001	2.93 (2.22 - 3.86)
		TT	145	16	<0.0001	12.99 (7.51 - 22.49)
	Indel -EU427539 (*ARMS2*)	Wt/Wt	190	269	-	Ref
		Wt/Indel	306	137	<0.0001	3.16 (2.41 - 4.16)
		Indel/Indel	119	15	<0.0001	11.23 (6.36 – 19.82)
	rs11200638 (*HTRA1*)	GG	194	271	-	Ref
		GA	292	138	<0.0001	2.96 (2.25 – 3.89)
		AA	130	17	<0.0001	10.68 (6.24 – 18.29)
Indian	rs10490924 (*ARMS2*)	GG	39	85	-	Ref
		GT	84	94	0.004	1.95 (1.21 – 3.15)
		TT	99	26	<0.0001	8.30 (4.67 – 14.74)
	Indel -EU427539 (*ARMS2*)	Wt/Wt	44	84	-	Ref
		Wt/Indel	79	88	0.017	1.71 (1.16 – 2.75)
		Indel/Indel	104	34	<0.0001	5.84 (3.43 – 9.94)
	rs11200638 (*HTRA1*)	GG	44	61	-	Ref
		GA	70	67	0.01	1.45 (0.87 – 2.42)
		AA	84	17	<0.0001	6.85 (3.58 – 13.12)

Homozygosity of the indel and the other variants were strongly associated with an increased risk of AMD in both the Indian and Australian cohorts. Combined homozygosities at the A69S and the rs11200638 along with the indel variant did not alter the risk of AMD significantly either in the Indian (OR=7.69, 95%CI, 4.07–14.51) or Australian cohorts (OR=10.61, 95%CI, 7.05–15.96).

**Figure 1:**
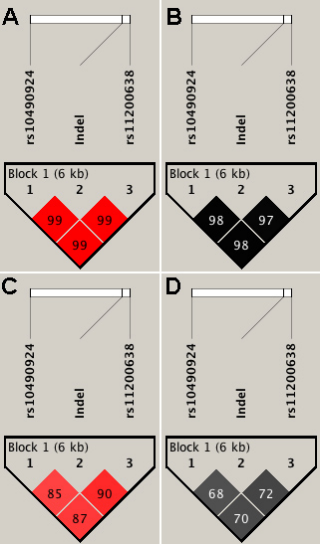
Linkage disequilibrium plots showing the three variants in the Australian and Indian cohorts. The D’ and r^2^ values between the SNPs are indicated inside the quadrants for the Australian (**A** and **B**) and the Indian (**C** and **D**), respectively.

The measure of linkage disequilibrium (LD) between the A69S, del443ins54 and rs11200638 variants were remarkably high across this 10q26 region with relatively higher values in the Australian (D’=0.99; r^2^=0.98) compared to the Indian (D’=0.87; r^2^=0.71) cohorts (Figure 1). Two major haplotypes (frequency >5%) were identified across the three variants with “T-Indel-A” being the risk haplotype and “G-WT (wild-type)-G” being protective in the Indian and Australian cohorts, respectively (Table 3). Different pairwise haplotype combinations with either the ‘T’ or ‘A’ allele at A69S in presence of the del443ins54 or its wild-type form along with the ‘G’ or ‘A’ allele of rs11200638, did not substantially alter the p values or ORs observed for either the risk or protective haplotypes as opposed to when all three variants were assessed together, reinforcing the observation of the high LD between variants in these two genes (Table 3).

**Table 3:** Major haplotype frequencies at the three loci harboring the ARMS2 (A69S and del443ins54) and HTRA1 variants in the Indian and Australian cohorts

HAPLOTYPES (5’-3’)			SOUTH INDIAN COHORT (n=433)				AUSTRALIAN COHORT (n=1054)			
rs1094924 *(ARMS2)*	del443ins54 *(ARMS2)*	rs11200638 *(HTRA1)*	% Cases (N=227)	% Controls (N=206)	P values	OR (95%CI)	% Cases (N=624)	% Controls (N=430)	P values	OR (95%CI)
**T**	**Indel**	**A**	**57.8**	**30.3**	**5.70x10^-16^**	**3.16 (2.34-4.19)**	**44.1**	**20**	**6.33x10^-30^**	**3.15 (2.57-3.85)**
G	Wt*	G	33	55.6	2.35x10^-11^	0.39 (0.29-0.52)	55.3	79.8	1.02x10^-30^	0.31 (0.25-0.38)
G	-	G	35.8	59.5	4.49x10^-12^	0.38 (0.29-0.50)	55.4	79.8	1.43x10^-30^	0.31 (0.26-0.38)
**T**	**-**	**A**	**58.4**	**30.4**	**1.82x10^-16^**	**3.20 (2.42-4.25)**	**44.1**	**20.2**	**1.55x10^-29^**	**3.11 (2.55-3.81)**
**T**	**Indel**	**-**	**59.1**	**31.8**	**1.32x10^-15^**	**3.09 (2.34-4.10)**	**44.4**	**20**	**1.18x10^-30^**	**3.19 (2.61-3.91)**
G	Wt*	-	33.5	50.4	2.54x10^-13^	0.36 (0.27-0.47)	55.4	79.8	1.43x10^-30^	0.31 (0.26-0.38)
**-**	**Indel**	**A**	**50.8**	**31.8**	**2.41x10^-15^**	**3.06 (2.31-4.05)**	**44.1**	**20**	**6.33x10^-30^**	**3.15 (2.57-3.86)**
-	Wt*	G	35.5	59.1	5.04x10^-12^	0.38 (0.29-0.50)	55.3	79.8	1.02x10^-30^	0.31 (0.26-0.38)

Combinations of the risk alleles at the three loci are bolded; *Wt = wildtype

## Discussion

These data provide an independent replication of the association of the *ARMS2* del443ins54 variant in two cohorts, and to the best of our knowledge for the first time among South Indians with AMD. The strong association of the del443ins54 along with the A69S and rs11200638 variants in the Indian and Australian cohorts were consistent with that observed in other populations [6-10]. Haplotype analysis with these three variants indicated that inclusion of del443ins54 in the haplotype neither increased nor decreased the risk of AMD in either cohort (Table 3).

The *ARMS2* and *HTRA1* genes are in high LD in European populations and thus dissecting out the role of one gene over the other has proved difficult. The advantage of undertaking a comparative analysis of genetic variants in populations of differing ethnicities expands the genetic diversity available and may provide the opportunity of identifying a more defined but associated region for further study. The current study highlighted similar degrees of associations across these three variants despite a relatively lower LD between the A69S, del443ins54 and rs11200638 variants in the Indian compared to the Australian cohort. However, it did confirm the presence of stratification differences between ethnicities with the allele frequency of the Indel of del443ins54 in South Indians being higher at 0.63 in cases compared to that in European populations (0.36–0.53) but lower than other Asian populations (0.73–0.88). The allele frequency in cases is similar to that previously shown by us in the assessment of the A69S (0.63) and the rs11200638 (0.60) variants of the *ARMS2* and *HTRA1* genes respectively, in the South Indian cohort. Evidence of population stratification has also been observed in AMD studies of the protective *CFHR3–1* deletion, with the highest frequencies of the deleted allele being present in African populations (16%–20%) compared to Asians (<2%) [15,16].

The potential role of the *ARMS2* and *HTRA1* gene in AMD is still unclear but functional dissection of the effect of the rs11200638 promoter variant in the *HTRA1* gene has revealed that this variant resides within a putative transcription binding site for the factors AP2α and SRF (serum response factor) [3,4]. Initial investigation of the influence of the homozygous risk genotype on *HTRA1* expression levels revealed consistently higher levels of expression with the AA genotype compared to the GG genotype [3,4]. In contrast, other studies of the rs11200638 variant have revealed no functional effect on *HTRA1* expression [2,6,17].

Analysis of the chromosome 10q26 risk haplotype inclusive of the *ARMS2* del443ins54 indel found decreased *ARMS2* expression and almost 3.0 fold increase in *HTRA1* expression [18]. Interestingly, a subsequent study has shown that while the *ARMS2* risk del443ins54 results in decrease in mRNA transcription levels of the *ARMS2* gene, a non risk associated variant (rs2736911) also leads to significantly reduced *ARMS2* transcript levels suggesting that *ARMS2* protein deficiency alone is unlikely to be pathogenic in AMD [17]. A functional role for *ARMS2* in mitochondrial homeostasis has also been suggested and the biology concerning mitochondrial dysfunction and the effects on age supports this notion [2,5]. However, subsequent immunofluorescence and immunoblot experiments localized *ARMS2* in retinal epithelial ARPE-19 cells and COS7 transfected cells to the cytosol rather than the mitochondria suggesting that *ARMS2* may not confer risk to AMD through the mitochondrial pathway [19]. Studies concerning the effects of AMD risk variants on *HTRA1* expression are equivocal and further investigations on the functional role of these variants are required.

In conclusion, we provide convincing evidence for the association of the del443ins54 variant with AMD, despite differences in allele, genotype and haplotype frequencies and LD across the 10q26 region reflecting population stratification differences in two different ethnicities. AMD, a complex multi-factorial disease is associated with multiple genomic regions with varying magnitudes of effect and the relevance of genetic associations differ between populations. Further, elucidation of the genetic basis of this disease through the analysis of individuals from different ethnic groups has the potential to provide useful insights into the genetic diversity of risk and protective variants within a gene as well as their contributions to disease. Also, meaningful genetic dissection of the *ARMS2* and *HTRA1* gene in this region will require much larger patient cohorts than have currently been assessed, or through the identification of other ethnic populations which show relatively lower levels of LD over this 10q26 region.
